# “Righteous Minds” in Health Care: Measurement and Explanatory Value of Social Intuitionism in Accounting for the Moral Judgments in a Sample of U.S. Physicians

**DOI:** 10.1371/journal.pone.0073379

**Published:** 2013-09-04

**Authors:** Jon C. Tilburt, Katherine M. James, Sarah M. Jenkins, Ryan M. Antiel, Farr A. Curlin, Kenneth A. Rasinski

**Affiliations:** 1 Division of General Internal Medicine, Mayo Clinic, Rochester, Minnesota, United States of America; 2 Biomedical Ethics Research Unit, Mayo Clinic, Rochester, Minnesota, United States of America; 3 Mayo Program in Professionalism and Ethics, Mayo Clinic, Rochester, Minnesota, United States of America; 4 Biomedical Statistics and Informatics, Mayo Clinic, Rochester, Minnesota, United States of America; 5 Department of Surgery, Mayo Clinic, Rochester, Minnesota, United States of America; 6 Department of Medicine, MacLean Center for Clinical Medical Ethics, University of Chicago, Chicago, Illinois, United States of America; 7 Department of Medicine, University of Chicago, Chicago, Illinois, United States of America; University of Colorado, United States of America

## Abstract

The broad diversity in physicians’ judgments on controversial health care topics may reflect differences in religious characteristics, political ideologies, and moral intuitions. We tested an existing measure of moral intuitions in a new population (U.S. physicians) to assess its validity and to determine whether physicians’ moral intuitions correlate with their views on controversial health care topics as well as other known predictors of these intuitions such as political affiliation and religiosity. In 2009, we mailed an 8-page questionnaire to a random sample of 2000 practicing U.S. physicians from all specialties. The survey included the Moral Foundations Questionnaire (MFQ30), along with questions on physicians’ judgments about controversial health care topics including abortion and euthanasia (no moral objection, some moral objection, strong moral objection). A total of 1032 of 1895 (54%) physicians responded. Physicians’ overall mean moral foundations scores were 3.5 for harm, 3.3 for fairness, 2.8 for loyalty, 3.2 for authority, and 2.7 for sanctity on a 0–5 scale. Increasing levels of religious service attendance, having a more conservative political ideology, and higher sanctity scores remained the greatest positive predictors of respondents objecting to abortion (β = 0.12, 0.23, 0.14, respectively, each p<0.001) as well as euthanasia (β = 0.08, 0.17, and 0.17, respectively, each p<0.001), even after adjusting for demographics. Higher authority scores were also significantly negatively associated with objection to abortion (β = −0.12, p<0.01), but not euthanasia. These data suggest that the relative importance physicians place on the different categories of moral intuitions may predict differences in physicians’ judgments about morally controversial topics and may interrelate with ideology and religiosity. Further examination of the diversity in physicians’ moral intuitions may prove illustrative in describing and addressing moral differences that arise in medical practice.

## Introduction

In their daily practice, physicians may face a multitude of ethically and morally complex situations that require action. As such, physicians’ moral beliefs play an important role in their professional practice. Generally speaking, surveys assessing the spectrum of physicians’ personal opinions about ethical dilemmas illustrate the degree of disunity among physicians on a range of contentious topics. [1], [2], [3], [4] But from where exactly do physicians’ diverse moral judgments about controversial aspects of clinical practice originate? And why might equally intelligent and conscientious professionals differ on moral issues in health care, particularly on topics like abortion and euthanasia?

The fields of social and cognitive psychology have recently generated novel approaches for defining basic differences in moral intuitions. Social-intuitionist theory posits that, at its core, moral reasoning follows moral intuitions, or “gut instincts”. Ideological divides do not therefore arise first from differences in moral reasoning, per se, but rather from deeper differences in innate intuitions, described by Haidt et al. as “moral foundations.” [5].

Haidt et al. have identified five dimensions of these intuitions, which make up the five moral foundations: *harm/care* (underlying the virtues of kindness, gentleness, and nurturance), *fairness/cheating* (generates our ideas of justice, rights, and autonomy), *loyalty/betrayal* (underlying the virtues of patriotism and self-sacrifice), *authority/subversion* (underlying the virtues of leadership, deference to legitimate authority, and respect for traditions), and *sanctity/degradation* (underlying the notion that the body can be desecrated/contaminated by immoral activities). Their research characterizes and quantifies how each of these intuitions work in areas of moral controversy. [6], [7] There are linear effects across the spectrum of each of the moral foundations associated with being socially liberal/conservative. At their extremes, very liberal respondents rely disproportionately on harm and fairness, while very conservative respondents rely fairly equally on all foundations.

In addition to political ideology, religious characteristics have also been shown to explain variances in how physicians make judgments about morally controversial health care topics. Previous studies in both the U.S. and internationally have established that, for physicians, religious characteristics influence moral decisions encountered in regular practice. [2], [3], [8], [9], [10], [11] Moreover, the public may associate intense religiosity with rash and extreme, or even visceral moral reactions – not unlike aspects of social intuitionism. However, the precise nature of the relationship between religiosity and moral intuitions as rigorously measured constructs has not been well defined, particularly among physicians. Add to this the general dearth of data on physicians’ political affiliations, and one can begin to see how little is known regarding the causes of diverse moral judgments in the medical profession. In his recent book on the topic, Haidt addresses why one might hypothesize that a lower degree of religiosity would be associated with higher relative ratings of *harm* and *fairness* intuitions as well as with liberal ideology. [12].

While the Moral Foundations Questionnaire has been tested in the general U.S. population, it has yet to be used in a sample of health professionals where morally controversial questions frequently arise. Given the diversity of physicians’ views on morally controversial topics they may encounter in their practice, this is an important population in which to begin to understand the impact that varied moral foundations may have on physicians’ divergent opinions and how those intuitions inter-relate with political ideology and religiosity.

We therefore sought to measure moral intuitions using an existing instrument (MFQ30), to assess the instrument’s consistency and explanatory utility in a new population, and to determine whether and how physicians’ moral intuitions are associated with their judgments about controversial health care topics as well as other known predictors of these intuitions such as religiosity and political affiliation. This study, the first of its kind to examine the moral foundations in the context of medical practice, serves as an important model for using the empirically-based, theoretically rigorous tools of social psychology to understand and inform important ethical debates in medicine.

## Materials and Methods

### Ethics Statement

This study was approved by the Mayo Clinic Institutional Review Board.

### Data Sharing Statement

A complete de-identified dataset is available from the corresponding author upon request.

### Study Participants and Data Collection

The methods have been described in detail previously. [13] In May of 2009, we mailed an 8-page, self-administered survey to 2000 practicing U.S. physicians randomly selected from the American Medical Association (AMA) Physician Masterfile, a database designed to include all licensed U.S. physicians. [14] Our sample included all listed physicians age 65 and under representing all specialties, excluding residents and those whose primary specialty was listed as “administration” only.

### Survey Instrument

Through an iterative process of literature review, question formulation, cognitive interviewing with physicians, and question revision**,** we developed the above survey containing questions about physicians’ perspectives on a variety of complex and controversial health care topics. The questionnaire included the MFQ30– the scale used to measure 5 moral intuitions – with minor adaptations to its stem language [from “Please read the following sentences and indicate your agreement or disagreement” (original) to “Please indicate your degree of agreement or disagreement with the following statements based on your initial reaction” (revised); and from “When you decide whether something is right or wrong, to what extent are the following considerations relevant to your thinking” (original) to “Please indicate how relevant each of the following features are to you in determining whether or not something is right or wrong”(revised)]. We also included questions about physicians’ demographic (i.e. age, gender, region of practice, specialty) and religious characteristics, as well as their primary political affiliation. (Full instrument available in Appendix S1) These demographic variables were selected to ascertain the general characteristics of our sample as well as based upon the *a priori* hypothesis that physicians’ age, gender, region of practice, and specialty were characteristics that might be associated with their responses to items in our survey.

Religiosity was assessed by asking, “How often do you attend religious services?” with nine response categories ranging from “Never” to “Several times a week”. Respondents self-identified their primary political affiliation (hereafter referred to as ideology) by selecting from the following response categories: “liberal”, “moderate”, “conservative”, or “other”. For analysis purposes, we categorized physician specialties into the following categories: “non-clinical”, “non-surgical procedural”, “non-surgical non-procedural”, “surgery”, “primary care”, and “other”.

We selected two morally controversial health care topics from the larger survey for this analysis: abortion and euthanasia. We asked physicians’ to rate their degree of moral objection to “abortion because the fetus has a chromosomal defect” and “helping a terminally ill patient to actively hasten his/her own death.”

### Management and Analysis of Data

Mailed paper survey responses were double entered and imported into SAS version 9.2 (SAS Institute, Cary, NC). We utilized the Response Rate 2 (RR2) definition from the American Association for Public Opinion Research Standard definitions when calculating our overall response rate. [15] Demographic differences between respondents and non-respondents (including sex, age, region of practice, and specialty) were assessed using Pearson chi-square tests.

Based on physicians’ responses to items included in the MFQ30, we calculated physicians’ mean scores in each of the five moral foundations: *harm/care, fairness/cheating, loyalty/betrayal, authority/subversion, sanctity/degradation*. A physician’s overall mean score for each foundation was calculated by averaging his or her responses to six survey items that together comprise that foundation’s subscale. All items had response categories that were scored on a 6-point scale ranging from 0 (strong disagreement with item or not relevant to moral decision-making) to 5 (strong agreement with item or extremely relevant to moral decision-making). Mean scores were not calculated for respondents with greater than 2 missing items among the six comprising each foundation. We assessed the internal consistency of each moral foundation with Cronbach’s alpha.

Associations between the moral foundations and demographics (age, gender, religiosity, and ideology) with level of moral objection to each scenario (abortion and euthanasia) were examined with linear regression models. For each model, the dependent variable was level of moral objection (0 = no objection, 1 = moderate objection, 2 = strong objection). Each predictor was modeled continuously, with gender modeled using binary coding (1 = male, 0 = female). Religiosity ranged from 1 (no attendance) to 9 (several times per week). Ideology ranged from 1 (liberal) to 3 (conservative), with the ‘other’ category excluded from these analyses. All analyses were performed using SAS version 9.2 (Cary, NC). P-values less than 0.01 were considered statistically significant. All results presented here are from unweighted analyses.

## Results

Of 2000 physicians, 105 could not be contacted; 1032/1895 eligible physicians returned completed surveys (AAPOR RR2 of 54%). [15] Characteristics of physician respondents are shown in Table 1. The majority of respondents were male (72%), 50 years of age or older (53%), and white (78%). Response rates varied somewhat by region (Northeast, 53%; South, 52%; Midwest, 62%; West, 52%; P = 0.03) and age category (<50 years, 51%; ≥50 years, 59%; P<0.001) but not by sex or specialty.

**Table 1:** Characteristics of the 1032 U.S. physician survey respondents.

Characteristic	No. (%)
Female sex	283 (28)^a^
Age (years)	
Less than 50	471 (47)
50 or older	540 (53)
Race or ethnic group	
White or Caucasian	786 (78)
Asian	146 (14)
Other	50 (5)
Black or African-American	25 (2)
American Indian or Alaska Native	4 (0.4)
Region^b^	
South	331 (32)
Midwest	251 (24)
Northeast	227 (22)
West	206 (20)
Primary Specialty	
Primary Care	407 (39)
Surgery	212 (21)
Procedural Specialty	206 (20)
Nonprocedural Specialty	175 (17)
Non-Clinical	22 (2)
Other	10 (1)
Political Ideology	
Moderate	426 (42) c
Conservative	291 (29)
Liberal	281 (28)
Other	21 (2)

Percentages shown are based on a denominator of 1032 unless otherwise noted.

a Sex, age, and race information available for *n* = 1011 respondents.

b 8 responding physicians were from Puerto Rico, and 9 were from the Pacific region (Alaska, Hawaii).

c Political affiliation data available for *n* = 1019 respondents.

### Moral Foundations–Means and Internal Consistency (Table 2)

Physicians’ overall mean scores for the five moral foundations ranged from 2.7 in the *sanctity* foundation to 3.5 in the *harm* foundation. There was, however, considerable variability in overall mean scores for individual items comprising the scales for each foundation (Table 2). Cronbach’s alpha scores for the five moral foundations showed moderate levels of internal consistency, ranging from 0.57 for the *harm* foundation to 0.83 for the *sanctity* foundation.

**Table 2:** Characteristics of responses for each item used in calculation of the 5 moral foundations.

Moral Foundation	Mean	SD	Range	No. missing	Cronbach’s Alpha^a^
**Harm**					
Overall (average of items)	3.5	0.8	0.8–5	19	0.57
Stem: Indicate your degree of agreement or disagreement with the following statements					
v32. Compassion for those who are suffering is the most crucial virtue.	4.1	1.0	0–5	19	
V38. One of the worst things a person could do is hurt a defenseless animal.	3.7	1.5	0–5	15	
V43. It can never be right to kill a human being.	2.5	1.9	0–5	20	
Stem: Indicate how relevant each of the following are in determining whether something is right or wrong. Whether or not someone…					
V48. Suffered emotionally.	3.3	1.2	0–5	20	
V54. Cared for someone weak or vulnerable.	3.3	1.3	0–5	26	
V59. Is cruel.	4.2	1.1	0–5	23	
**Fairness**					
Overall (average of items)	3.3	0.7	0. 7–5	19	0.62
Stem: Indicate your degree of agreement or disagreement with the following statements					
V33. When the government makes laws, the number one principle should be ensuring that everyone is treated fairly.	3.7	1.4	0–5	22	
V39. Justice is the most important requirement for a society.	3.7	1.0	0–5	17	
V44. I think it’s morally wrong that rich children inherit a lot of money while poor children inherit nothing.	1.2	1.4	0–5	18	
Stem: Indicate how relevant each of the following are in determining whether something is right or wrong. Whether or not someone…					
V49. Was treated differently than others.	3.3	1.2	0–5	20	
V55. Acts unfairly.	3.6	1.2	0–5	23	
V60. Denies others their rights.	4.3	1.0	0–5	26	
**Loyalty**					
Overall (average of items)	2.8	0.8	0.5–5	19	0.62
Stem: Indicate your degree of agreement or disagreement with the following statements					
V34. I am proud of my country’s history.	3.9	1.4	0–5	17	
V40. People should be loyal to their family members, even if they have done something wrong.	2.8	1.5	0–5	19	
V45. It is more important to be a team player than to express one’s self.	2.0	1.4	0–5	18	
Stem: Indicate how relevant each of the following are in determining whether something is right or wrong. Whether or not someone…					
V50. Shows love for his or her country	2.2	1.5	0–5	22	
V56. Did something to betray his or her group	3.2	1.3	0–5	28	
V61. Shows a lack of loyalty.	2.9	1.3	0–5	26	
**Authority**					
Overall (average of items)	3.2	0.8	0.7–5	20	0.67
Stem: Indicate your degree of agreement or disagreement with the following statements					
V35. Respect for authority is something all children need to learn.	4.3	0.9	0–5	17	
V41. Men and women each have different roles to play in society.	3.0	1.6	0–5	21	
V46. If I were a soldier and disagreed with my commanding officer’s orders, I would obey anyway because that is my duty.	2.9	1.5	0–5	14	
Stem: Indicate how relevant each of the following are in determining whether something is right or wrong. Whether or not someone…					
V51. Shows a lack of respect for authority.	2.9	1.4	0–5	23	
V57. Conforms to the traditions of society.	2.2	1.3	0–5	26	
V62. Causes chaos or disorder.	3.4	1.3	0–5	23	
**Sanctity**					
Overall (average of items)	2.7	1.2	0–5	22	0.83
Stem: Indicate your degree of agreement or disagreement with the following statements					
V36. People should not do things that are disgusting, even if no one is harmed.	3.0	1.7	0–5	23	
V42. I would call some acts wrong on the grounds that they are unnatural.	2.4	1.7	0–5	29	
V47. Chastity is an important and valuable virtue.	2.9	1.6	0–5	33	
Stem: Indicate how relevant each of the following are in determining whether something is right or wrong. Whether or not someone…					
V52. Violates standards of purity and decency.	2.9	1.5	0–5	28	
V58. Does something disgusting.	2.3	1.5	0–5	26	
V63. Acts in a way that God would approve of.	2.6	1.9	0–5	35	

a Raw Cronbach’s alpha scores are reported.

Response categories ranged from 0 (strongly disagree/not at all relevant) to 5 (strongly agree/extremely relevant).

### Associations between Moral Foundations, Physician Characteristics, and Moral Judgments (Table 3)

Associations between respondent characteristics and moral objection were assessed with linear regression models. The coefficients that follow can be interpreted as the estimated change in average objection level for each one-unit increase in the predictor.

In a model including age, gender, religiosity, and ideology, both religiosity and ideology were positively associated with objecting to abortion (β = 0.15 & 0.28, respectively, each p<0.001) as well as euthanasia (β = 0.11 & 0.23, respectively, each p<0.001). In other words, a physician’s average moral objection level increases with greater religious attendance and more conservative ideology.

In another model including the five moral foundations as predictors, *fairness* and *authority* were significantly negatively associated with objection to abortion [β = −0.21 (p<0.001) & −0.13 (p<0.01), respectively], while *sanctity* was found to have a significantly positive effect (β = 0.34, p<0.001). In a model examining associations between moral foundations and objection to euthanasia, only *sanctity* was found to be significantly positively associated with moral judgments about this topic (β = 0.31, p<0.001). (See Models 1 and 2 in Table 3).

**Table 3:** Predicting moral objection ratings about abortion and euthanasia from physician demographics and moral foundations (three separate models for each), *N = *1032.

	Moral Objection to Abortion			Moral Objection to Euthanasia		
Mean (SD)	0.65 (0.80)			1.0 (0.81)		
	*Regression coefficients*			*Regression coefficients*		
*Predictors*	Model 1†	Model 2†	Model 3	Model 1†	Model 2†	Model 3
Age	−0.006		−0.006	0.003		0.0007
Gender (ref = female)	0.06		0.10	−0.06		−0.03
Religious attendance	0.15**		0.12**	0.11**		0.08**
Political ideology	0.28**		0.23**	0.23**		0.17**
Harm		0.04	0.08		0.07	0.10
Fairness		−0.21**	−0.08		−0.11	−0.03
Loyalty		0.03	0.009		−0.03	−0.05
Authority		−0.13*	−0.12*		−0.06	−0.07
Sanctity		0.34**	0.14**		0.31**	0.17**

NOTE: Objection to abortion and euthanasia ranged from 0–2 in direction of degree of moral objection (none, moderate, strong). Ideology ranged 1–3 in direction of increasing conservatism (i.e. liberal, moderate, conservative). Religious attendance ranged 1–9 in direction of increasing attendance. Each predictor is modeled continuously with the exception of gender (coded such that 0 =  female and 1 =  male). Due to missing data, the exact *N* for each item varied (*N = *923 for abortion; *N* = 981 for euthanasia). Additional results with interactions presented in text only.

† To be compared to Koleva et al. (2012).

* p<0.01.

** p<0.001.

In single multivariate models that combined demographic and moral foundation variables using objection to abortion and objection to euthanasia, respectively, as the dependent variables, most factors reported above remained significant predictors of respondents’ objections to abortion and euthanasia. Increasing levels of religious service attendance, having a more conservative political ideology, and higher *sanctity* scores remained the greatest positive predictors of respondents objecting to abortion (β = 0.12, 0.23, 0.14, respectively, each p<0.001) as well as euthanasia (β = 0.08, 0.17, and 0.17, respectively, each p<0.001). Higher authority scores were also significantly negatively associated with objection to abortion (β = −0.12, p<0.01), but not euthanasia. Although the *fairness* intuition appeared significantly negatively associated with objections to abortion in models without demographics, religious attendance, and political ideology, it was no longer significant in the multivariate model. (See Model 3 in Table 3).

To assess whether physician demographics (age, gender, religiosity, or ideology) modify the effects of the moral foundations on objection level, we tested for interactions between the demographics and foundations, as well as between the demographics themselves. No significant interactions were found with respect to moral objection for euthanasia.

For abortion, however, we found that religiosity interacted significantly with *sanctity* as well as ideology. Moreover the effect of *sanctity* strengthens as religious attendance increases. For those with no religious attendance, *sanctity* has only a small effect on objection (β = 0.05, p = 0.17), while for those at the other end of the spectrum (attendance several days per week), the effect is much stronger (β = 0.26, p<0.001).

In addition to this, the effect of ideology is stronger as religious attendance increases. For those with no religious attendance, the effect of ideology is very slight (β = 0.07, p = 23), while for those with very frequent religious attendance, the effect is strong – being more conservative is associated with stronger objection to abortion (β = 0.43, p<0.001).

## Discussion

In this survey of U.S. physicians’ moral judgments about controversial health care topics and the composition of their moral intuitions, we found the MFQ30 to be a potentially useful explanatory tool for explaining differences in physicians’ moral judgments. We also found significant differences in the relative weight given to those foundations based on physicians’ self-identified political views and religiosity in a manner largely consistent with Moral Foundation Theory. Where there are clear culture war debates, among the five moral foundations measured, *sanctity* was most consistently and strongly associated with physicians’ views on abortion and euthanasia. Like others studying culture wars in the general population [7], we found that *sanctity* intuitions seem to play a strong role in physicians’ judgments on par with (and apparently intertwined with) religiosity and politics. Other intuitions seem to play a less influential role.

For the particular moral judgments investigated, abortion and euthanasia, religious institutions have played a prominent role in public debate, making religiosity a plausible predictor of objection. For these analyses intensity of religiosity as a measured construct may be more valid (i.e. psychometrically coherent) than self-described “conservatism” – which may encompass multiple connotations including economic, geopolitical, as well as social meanings. Despite heterogeneity in the meaning of “conservative”, self-described political affiliation remains a strong predictor of objections to the controversial health care topics under consideration in this analysis and may be attributable in part to deeper moral intuitions such as *sanctity*.

Some associations uncovered in this analysis are puzzling. For example, higher *authority* scores were associated with less (not more) objection to abortion. We speculate, but cannot demonstrate that the legally sensitive health care practices queried in our survey may have elicited an “anti-authority” impulse (vis-à-vis state power) among self-described socially conservative respondents who otherwise might be very accepting of authority generally. For instance, to strongly oppose the legal practice of abortion currently in the US could be construed as a subversive or anti-(state) authority position. In the case of euthanasia, ideology and religiosity were similarly directly associated with objection in directions consistent with Moral Foundations Theory.

Why would physicians who scored higher on *fairness* concerns be less likely to object to abortion, at least in unadjusted analyses? Our finding suggests that physicians who do not object to abortion may be relatively more focused on fairness, and conversely, physicians who object to abortion may be somewhat less focused on fairness relative to other potentially relevant categories of moral intuition, but such effects do not appear to be robust after accounting for demographic, religious, and political differences.

These findings corroborate the potential role for Moral Foundations Theory in explaining the diversity of physician judgments in matters of moral controversy in the profession, particularly when matters related to *sanctity* are at stake. Koleva et al found that *fairness* and *loyalty* were each associated with only one of 20 issues of significance in culture wars, *authority* with three of 20, *harm* intuitions with five of 20, whereas *sanctity* was associated with 10 of 20 culture war-related issues. [7] Analogously, we seem to have identified a strong role for *sanctity* as a driver of physicians’ moral judgments, albeit in a much more circumscribed set of judgments.

Our approach has several limitations. In surveys like this one, there is always the possibility of non-response bias. Beyond the modest demographic differences found between respondents and non-respondents, there may have been unmeasured differences between respondent and non-respondent groups which we were unable to account for. Furthermore, our sample of just 1032 physicians, though selected randomly so as to minimize bias and increase the generalizability of our findings, is nevertheless just a snapshot of the 661,400 physicians practicing in the U.S. according to 2010 census data. In addition, while the AMA Physician Masterfile is the most comprehensive listing of US physicians available, it relies on physician self-report for key practice characteristics. For instance, specialty data listed in the AMA Masterfile includes self-reported information that is not verified with specialty boards. The descriptive statistics reported here therefore may not fully reflect all US physician opinion.

Furthermore, based on the Cronbach’s alpha statistics calculated for the five moral foundation measures utilized in our survey, the internal consistency of the items comprising each foundation was found to be moderate at best; our inferences about the nature of the associations measured in this study must therefore be weighed in light of this weakness. However, it should be noted that the Cronbach’s alpha statistics reported here do not differ greatly from those reported in Koleva et al. [7] We also opted to not provide respondents the opportunity to select “uncertain” or “don’t know” in all survey items using Likert response scales. We acknowledge this inherent limitation in our ability to ascertain the presence of neutral or acquiescent bias in our study; however, there is continued disagreement in opinion research about the role of “uncertain” categories in response scales. The so-called “forced choice” scale, as we chose to utilize, is one acceptable approach. [16], [17] Finally, our choice of a 3-level, uni-dimensional categorization of political ideology may have limited our ability to explain associations between ideology and moral foundations in a more nuanced manner.

Despite these limitations, utilizing social intuitionist theory in this study did help to describe variance in physicians’ moral judgments about controversial practices. This or other similar theoretical models may be fruitful approaches with which to describe with better precision the nature of disagreement about controversial medical topics among a population of influential professionals. Fairly characterizing the opponent’s position and understanding the deep drivers of moral discord in medicine and public life may be important first steps in devising more constructive dialogue about the merits of differing positions in a pluralistic society. Moral Foundations Theory and similar approaches to moral psychology may contribute to civil, dialogue on matters of moral controversy in the medical profession and public life.

## Supporting Information

Appendix S1 **Survey instrument.**
(PDF)

## References

[1] Craig A, Cronin B, Eward W, Metz J, Murray L, et al (2007) Attitudes toward physician-assisted suicide among physicians in Vermont. J Med Ethics 33: 400–403. 17601867 10.1136/jme.2006.018713 PMC2598148

[2] Curlin FA, Lawrence RE, Chin MH, Lantos JD (2007) Religion, conscience, and controversial clinical practices. N Engl J Med 356: 593–600. 17287479 10.1056/NEJMsa065316 PMC2867473

[3] Lawrence RE, Curlin FA (2009) Physicians’ beliefs about conscience in medicine: a national survey. Acad Med 84: 1276–1282. 19707071 10.1097/ACM.0b013e3181b18dc5 PMC2859045

[4] Lawrence RE, Rasinski KA, Yoon JD, Curlin FA (2011) Obstetrician-gynecologists’ views on contraception and natural family planning: a national survey. Am J Obstet Gynecol 204: 124 e121–127. 10.1016/j.ajog.2010.08.051 PMC3052964 21074134

[5] Haidt J (2007) The new synthesis in moral psychology. Science 316: 998–1002. 17510357 10.1126/science.1137651

[6] Graham J, Nosek BA, Haidt J, Iyer R, Koleva S, et al (2011) Mapping the moral domain. J Pers Soc Psychol 10(2): 366–385. 10.1037/a0021847 PMC3116962 21244182

[7] Koleva S, Graham J, Ditto P, Iyer R, Haidt J (2012) Tracing the threads: how five moral concerns (especially Purity) help explain culture war attitudes. Journal of Research in Personality 46: 184–194.

[8] Combs MP, Antiel RM, Tilburt JC, Mueller PS, Curlin FA (2011) Conscientious refusals to refer: findings from a national physician survey. J Med Ethics 37: 397–401. 21335574 10.1136/jme.2010.041194 PMC4019379

[9] Aghababaei N, Wasserman JA (2013) Attitude Toward Euthanasia Scale: Psychometric Properties and Relations With Religious Orientation, Personality, and Life Satisfaction. Am J Hosp Palliat Care. doi: 10.1177/1049909112472721. 10.1177/1049909112472721 23349343

[10] Bulow HH, Sprung CL, Baras M, Carmel S, Svantesson M, et al (2012) Are religion and religiosity important to end-of-life decisions and patient autonomy in the ICU? The Ethicatt study. Intensive Care Med 38: 1126–1133. 22527070 10.1007/s00134-012-2554-8

[11] Pawlikowski J, Sak JJ, Marczewski K (2012) Physicians’ religiosity and attitudes towards patients. Ann Agric Environ Med 19: 503–507. 23020047

[12] Haidt J (2012) The Righteous Mind: Why Good People Are Divided by Politics and Religion. Toronto: Pantheon Books.

[13] Antiel RM, Curlin FA, James KM, Tilburt JC (2009) Physicians’ beliefs and U.S. health care reform–a national survey. N Engl J Med 361: e23. 19752464 10.1056/NEJMp0907876 PMC3745004

[14] American Medical Association (2013) AMA Physician Masterfile. Available: https://www.ama-assn.org/ama/pub/about-ama/physician-data-resources/physician-masterfile.page. Accessed 2013 Aug 2.

[15] The American Association for Public Opinion Research (2011) Standard Definitions: Final Dispositions of Case Codes and Outcome Rates for Surveys: AAPOR.

[16] Krosnick JA (1999) Survey research. Annu Rev Psychol 50: 537–567. 15012463 10.1146/annurev.psych.50.1.537

[17] Smyth J, Dillman D, Christian L, Stern M (2006) Comparing check-all and forced-choice question formats in web surveys. Public Opin Q 70: 66–77.

