# Supplementary material for: “Righteous Minds” in Health Care: Measurement and Explanatory Value of Social Intuitionism in Accounting for the Moral Judgments in a Sample of U.S. Physicians
Source: PLoS One. 2013 Sep 4;8(9):e73379. doi: 10.1371/journal.pone.0073379 (PMC3762735; doi:10.1371/journal.pone.0073379)
Supplement: Appendix S1 — Survey instrument. (PDF) [file pone.0073379.s001.pdf]

APPENDIX S1

Moral Beliefs & Clinical Practice: A National Physician Survey

1-4

INSTRUCTIONS: PLEASE CHECK THE APPROPRIATE BOX OR FILL IN THE BLANK AS INDICATED.

SECTION A: SOURCES OF MORAL GUIDANCE

1. When you were a medical student, did your school conduct a physicians' oath ceremony?

5_ 1☐ No 2☐ Yes 3☐ I cannot recall

↓ (If Yes)

6_ **Did you participate in the ceremony?** 1☐ No 2☐ Yes

As best you can recall, which oath did you take (choose one)?

7_ 1☐ Hippocratic Oath (either original or modified)
2☐ Declaration of Geneva (either original or modified)
3☐ Oath/Prayer of Maimonides
4☐ Student/faculty authored oath
5☐ Osteopathic Oath
6☐ Other (please indicate): ______________________________
7☐ I cannot recall

2. How much do you think physicians' oaths have influenced your professional life?

8_ 1☐ A lot
2☐ Somewhat
3☐ Not very much
4☐ Not at all

3. Besides physicians' oaths, what other sources of moral guidance, if any, would you say have <u>significantly</u> influenced your professional practice? (Mark all that apply.)

9_ 1☐ The AMA Code of Ethics
2☐ Other professional society codes, please specify: ______________________
3☐ Great moral teachers, please specify: ______________________
4☐ Specific traditions, please specify: ______________________
5☐ Your personal sense of right and wrong
6☐ Other, please specify: ______________________

SECTION B: ETHICAL PRINCIPLES

4. Which of the following ethical principles is the <u>most</u> important in your practice as a physician? (Mark one.)

10_ 1 ☐ Respect for autonomy - *honoring the rights of patients to make decisions for themselves*

2 ☐ Justice - *seeking fair treatment of patients based on medical need and fair distribution of healthcare resources*

3 ☐ Beneficence/Non-maleficence - *promoting the well-being of patients and preventing illness, while minimizing harm*

SECTION C: YOUR PERSPECTIVE ON SOME COMPLEX HEALTHCARE TOPICS

5. Please indicate the degree to which you object (if at all), for moral reasons, to each of the following medical practices. Assume that all treatments mentioned <u>achieve their intended effect</u>.

			Strong moral objection	Moderate moral objection	No moral objection
11_	a.	Withholding a diagnosis from a patient because, in your judgment, telling him/her would hinder clinical improvement	2 ☐	1 ☐	0 ☐
12_	b.	Misrepresenting the truth to an insurance company in order for a patient to receive treatment you believe is medically necessary	2 ☐	1 ☐	0 ☐
13_	c.	Helping a terminally ill patient to actively hasten his/her own death	2 ☐	1 ☐	0 ☐
14_	d.	Abortion because the fetus has a chromosomal defect	2 ☐	1 ☐	0 ☐
15_	e.	Physician participation in capital punishment	2 ☐	1 ☐	0 ☐
16_	f.	Physician participation in aggressive military interrogation procedures	2 ☐	1 ☐	0 ☐
17_	g.	Collaborating with a traditional healer in a patient's care	2 ☐	1 ☐	0 ☐
18_	h.	Cosmetic surgery for enhancement of breast size or appearance	2 ☐	1 ☐	0 ☐
19_	i.	Helping a patient achieve a medical or surgical sex change	2 ☐	1 ☐	0 ☐
20_	j.	Paying unrelated donors for their organs (assuming the payment is nonexploitative)	2 ☐	1 ☐	0 ☐
21_	k.	Restricting access to organ transplants for patients older than 65 years	2 ☐	1 ☐	0 ☐
22_	l.	Prescribing a very expensive medication for off-label use	2 ☐	1 ☐	0 ☐
23_	m.	Using cost-effectiveness data to determine which treatments will be offered to patients	2 ☐	1 ☐	0 ☐

SECTION D: YOUR VIEWS OF HUMAN DIGNITY

Read the following case and indicate which statement best reflects your view of the patient's dignity:

Three months ago, an 81-year old woman with a past history of moderate dementia suffered an acute left hemispheric stroke with residual dense right hemiparesis. Her course was complicated by an in-hospital fall resulting in hip fracture that could not be repaired and recurrent aspiration pneumonia leading to PEG tube placement. When awake, she is very uncomfortable. Despite aggressive inpatient rehabilitation, she is unable to participate in or enjoy any activities of daily living and does not remember her family. Her advance directive states she would never want to live in a chronically debilitated condition or in a nursing home.

Please rate your degree of agreement or disagreement with the following statements.

6. This patient's life is no longer worth living.

24_ 1 ☐ Strongly agree
2 ☐ Moderately agree
3 ☐ Moderately disagree
4 ☐ Strongly disagree

7. Please mark the statement you believe most closely reflects the current state of this patient's dignity.

25_ 1 ☐ This patient has lost all her dignity.
2 ☐ This patient has lost some, but retains a minimal level of her dignity.
3 ☐ This patient has full dignity.

8. There are many debates about human dignity in medical ethics and practice. Please indicate whether you agree or disagree with the following statements about human dignity:

			Strongly disagree	Moderately disagree	Moderately agree	Strongly agree
26_	a.	Dignity is a concept that has no practical relevance for clinical medicine	1 ☐	2 ☐	3 ☐	4 ☐
27_	b.	Patients' dignity comes from their ability to make significant choices about their lives	1 ☐	2 ☐	3 ☐	4 ☐
28_	c.	Sometimes humans lose all their dignity	1 ☐	2 ☐	3 ☐	4 ☐
29_	d.	Dignity is given to humans by a creator	1 ☐	2 ☐	3 ☐	4 ☐
30_	e.	All living humans have the same amount of dignity	1 ☐	2 ☐	3 ☐	4 ☐
31_	f.	Treating people with dignity means nothing more than treating them as they would want to be treated	1 ☐	2 ☐	3 ☐	4 ☐

SECTION E: YOUR MORAL BELIEFS

In the following section, we are interested in understanding some of your thoughts <u>about life in general</u>. Some items may seem odd or irrelevant.

9. Please indicate your degree of agreement or disagreement with the following statements based on your initial reaction.

			Strongly disagree	Moderately disagree	Slightly disagree	Slightly agree	Moderately agree	Strongly agree
32_	a.	Compassion for those who are suffering is the most crucial virtue. . .	0 ☐	1 ☐	2 ☐	3 ☐	4 ☐	5 ☐
33_	b.	When the government makes laws, the number one principle should be ensuring that everyone is treated fairly..	0 ☐	1 ☐	2 ☐	3 ☐	4 ☐	5 ☐
34_	c.	I am proud of my country's history.. .	0 ☐	1 ☐	2 ☐	3 ☐	4 ☐	5 ☐
35_	d.	Respect for authority is something all children need to learn.	0 ☐	1 ☐	2 ☐	3 ☐	4 ☐	5 ☐
36_	e.	People should not do things that are disgusting, even if no one is harmed.	0 ☐	1 ☐	2 ☐	3 ☐	4 ☐	5 ☐
37_	f.	It is better to do good than to do bad.	0 ☐	1 ☐	2 ☐	3 ☐	4 ☐	5 ☐
38_	g.	One of the worst things a person could do is hurt a defenseless animal.	0 ☐	1 ☐	2 ☐	3 ☐	4 ☐	5 ☐
39_	h.	Justice is the most important requirement for a society.	0 ☐	1 ☐	2 ☐	3 ☐	4 ☐	5 ☐
40_	i.	People should be loyal to their family members, even if they have done something wrong.	0 ☐	1 ☐	2 ☐	3 ☐	4 ☐	5 ☐
41_	j.	Men and women each have different roles to play in society.	0 ☐	1 ☐	2 ☐	3 ☐	4 ☐	5 ☐
42_	k.	I would call some acts wrong on the grounds that they are unnatural..	0 ☐	1 ☐	2 ☐	3 ☐	4 ☐	5 ☐
43_	l.	It can never be right to kill a human being.	0 ☐	1 ☐	2 ☐	3 ☐	4 ☐	5 ☐
44_	m.	I think it's morally wrong that rich children inherit a lot of money while poor children inherit nothing.. .	0 ☐	1 ☐	2 ☐	3 ☐	4 ☐	5 ☐

Continued on page 5...

			Strongly disagree	Moderately disagree	Slightly disagree	Slightly agree	Moderately agree	Strongly agree
45_	n.	It is more important to be a team player than to express oneself.	0 ☐	1 ☐	2 ☐	3 ☐	4 ☐	5 ☐
46_	o.	If I were a soldier and disagreed with my commanding officer's orders, I would obey anyway because that is my duty.	0 ☐	1 ☐	2 ☐	3 ☐	4 ☐	5 ☐
47_	p.	Chastity is an important and valuable virtue.	0 ☐	1 ☐	2 ☐	3 ☐	4 ☐	5 ☐

The next set of questions also relates to your moral beliefs about life in general.

10. Please indicate how relevant each of the following features are to you in determining whether or not something is right or wrong.

Whether or not someone. . .

			Not at all relevant	Not very relevant	Slightly relevant	Somewhat relevant	Very relevant	Extremely relevant
48_	a.	suffered emotionally.	0 ☐	1 ☐	2 ☐	3 ☐	4 ☐	5 ☐
49_	b.	was treated differently than others	0 ☐	1 ☐	2 ☐	3 ☐	4 ☐	5 ☐
50_	c.	shows love for his or her country	0 ☐	1 ☐	2 ☐	3 ☐	4 ☐	5 ☐
51_	d.	shows a lack of respect for authority.	0 ☐	1 ☐	2 ☐	3 ☐	4 ☐	5 ☐
52_	e.	violates standards of purity and decency	0 ☐	1 ☐	2 ☐	3 ☐	4 ☐	5 ☐
53_	f.	is good at math	0 ☐	1 ☐	2 ☐	3 ☐	4 ☐	5 ☐
54_	g.	cared for someone weak or vulnerable.	0 ☐	1 ☐	2 ☐	3 ☐	4 ☐	5 ☐
55_	h.	acts unfairly	0 ☐	1 ☐	2 ☐	3 ☐	4 ☐	5 ☐
56_	i.	did something to betray his or her group.	0 ☐	1 ☐	2 ☐	3 ☐	4 ☐	5 ☐
57_	j.	conforms to the traditions of society .	0 ☐	1 ☐	2 ☐	3 ☐	4 ☐	5 ☐
58_	k.	does something disgusting	0 ☐	1 ☐	2 ☐	3 ☐	4 ☐	5 ☐
59_	l.	is cruel.	0 ☐	1 ☐	2 ☐	3 ☐	4 ☐	5 ☐
60_	m.	denies others their rights	0 ☐	1 ☐	2 ☐	3 ☐	4 ☐	5 ☐
61_	n.	shows a lack of loyalty	0 ☐	1 ☐	2 ☐	3 ☐	4 ☐	5 ☐
62_	o.	causes chaos or disorder	0 ☐	1 ☐	2 ☐	3 ☐	4 ☐	5 ☐
63_	p.	acts in a way that God would approve of	0 ☐	1 ☐	2 ☐	3 ☐	4 ☐	5 ☐

SECTION F: PROFESSIONAL RESPONSIBILITY & SOCIETAL ISSUES

Please rate your degree of agreement or disagreement with the following statements.

11. I would favor limiting reimbursement for expensive drugs and procedures if that would help expand access to basic healthcare for those currently lacking such care.

64_ 1 ☐ Strongly agree
2 ☐ Moderately agree
3 ☐ Moderately disagree
4 ☐ Strongly disagree

12. Every physician is professionally obligated to care for the uninsured and underinsured.

65_ 1 ☐ Strongly agree
2 ☐ Moderately agree
3 ☐ Moderately disagree
4 ☐ Strongly disagree

13. Addressing societal health policy issues, as important as that may be, falls outside the scope of my professional obligations as a physician.

66_ 1 ☐ Strongly agree
2 ☐ Moderately agree
3 ☐ Moderately disagree
4 ☐ Strongly disagree

14. Physicians have a professional obligation to refer patients for all legal medical services for which the patients are candidates, even if the physician believes that such a referral is immoral.

67_ 1 ☐ Strongly agree
2 ☐ Moderately agree
3 ☐ Moderately disagree
4 ☐ Strongly disagree

SECTION G: YOUR RELIGIOUS & SPIRITUAL CHARACTERISTICS

For some physicians, their religious or spiritual views may be important to their moral beliefs.

15. To what extent do you consider yourself a spiritual person?

68_ 1 ☐ Very spiritual
2 ☐ Moderately spiritual
3 ☐ Slightly spiritual
4 ☐ Not spiritual at all

16. Which of the following best indicates your religious affiliation?

69-70_ 1 ☐ None
2 ☐ Buddhist
71_ 3 ☐ Hindu
4 ☐ Jewish →
5 ☐ Muslim
6 ☐ Roman Catholic
7 ☐ Eastern Orthodox
8 ☐ Protestant
72_ 9 ☐ Other Christian
10 ☐ Other religion (please specify):

____

Would you say you are... (if Jewish)

1 ☐ Orthodox
2 ☐ Conservative
3 ☐ Reform
4 ☐ Secular
5 ☐ Other

Do you consider yourself evangelical? (if Roman Catholic, Eastern Orthodox, Protestant, or Other Christian)

1 ☐ Yes 2 ☐ No

17. How often do you attend religious services?

73_ 1 ☐ Never
2 ☐ Less than once a year
3 ☐ About once or twice a year
4 ☐ Several times a year
5 ☐ About once a month
6 ☐ Two to three times a month
7 ☐ Nearly every week
8 ☐ Every week
9 ☐ Several times a week

18. How important would you say your religion is in your own life?

74_ 1 ☐ The most important part of my life
2 ☐ Very important in my life
3 ☐ Fairly important in my life
4 ☐ Not very important in my life
9 ☐ Not applicable, I have no religion

19. To what extent do you agree or disagree with the following statements?

		Agree strongly	Agree somewhat	Disagree somewhat	Disagree strongly
75_	a. There is truth in one religion	1 ☐	2 ☐	3 ☐	4 ☐
76_	b. Different religions have different versions of the truth, and each may be equally right in its own way	1 ☐	2 ☐	3 ☐	4 ☐
77_	c. There is no one, true, right religion	1 ☐	2 ☐	3 ☐	4 ☐

SECTION H: YOUR BACKGROUND

20. Do you consider yourself Hispanic or Latino?

78_ 1 ☐ Yes 2 ☐ No

21. How do you classify your race? (Mark only one.)

79_ 1 ☐ Asian → **Do you think of yourself as ...**
- 1 ☐ East Asian or Pacific Islander
- 2 ☐ South Asian
- 3 ☐ Other Asian

2 ☐ Black or African-American

80_ 3 ☐ American Indian or Alaskan Native

4 ☐ White or Caucasian

5 ☐ Other ____________________

22. How would you characterize yourself on social issues?

81_ 1 ☐ Conservative

2 ☐ Moderate

3 ☐ Liberal

4 ☐ Other (please specify) ______________________________

Please let us know any other thoughts you have on the issues addressed in this questionnaire.

82_ ____

____

____

____

____

____

____

____

Please return this survey in the enclosed, postage-paid envelope.

THANK YOU FOR PARTICIPATING!

Section E: Used with the permission of Jonathan Haidt, Ph.D., University of Virginia.
http://faculty.virginia.edu/haidtlab/mft/index.php?t=questionnaires
